# Novel directions for G × E analysis in psychiatry

**DOI:** 10.1017/S2045796014000584

**Published:** 2014-10-08

**Authors:** A. A. E. Vinkhuyzen, N. R. Wray

**Affiliations:** The University of Queensland, Queensland Brain Institute, St Lucia, QLD 4072, Australia

**Keywords:** Gene–environment interaction, polygenic, psychiatric genetics

## Abstract

G × E in psychiatry may explain why environmental risk factors have big impact in some individuals but not in others, and conversely why relatives that are genetically at risk for disease do not all develop disease. Here we discuss two novel methods that use an aggregate genome-wide measure of genetic risk to detect G × E and estimate its effect in the population using data currently available and data we anticipate will be available in the near future. The first method exploits summary statistics from large-scale genome-wide association studies ignorant of the environmental conditions and detects G × E in an out-of-sample risk-profiling framework. The second method relies on larger samples and is based on a mixed linear model framework. It estimates variance explained directly from single nucleotide polymorphisms and environmental measures. Both methods have great potential to improve public health interventions focusing on risk-based screening that is informed by both genetic and environmental risk factors.

## Introduction

The importance of gene-by-environment interaction (G × E) in psychiatry has intuitive appeal and is often discussed. However, the dearth of data to investigate G × E means that empirical evidence is modest. Here we consider how the era of genome-wide genotyping opens new approaches for G × E research. G × E in disease aetiology implies environmental factors control disease outcome conditional on genetic predisposition, and conversely, genetic factors control disease outcome conditional on environmental exposure. It explains why genetic and environmental factors can have a big impact in some individuals but not in others. G × E research aims to identify non-marginal genetic and environmental effects, that is, effects of a specific genetic (or environmental) risk factor that does not act in the population as a whole when averaging over all other variables but only act conditional on an environmental (or genetic) variable.

Interpretation of G × E research requires careful consideration of the hypotheses tested, first recognising that presence of statistical interaction depends on – and may be induced by – the scale of measurement. In the past 15 years, the majority of G × E researches have used a molecular genetic approach focusing on candidate genes and specific environmental risk factors. Researchers hypothesised environmental factors controlled disease outcome only if a single genetic mutation was present (or vice versa, a genetic mutation to control disease outcome only under certain environmental circumstances). For complex genetic diseases including psychiatric disorders, the effect of a specific genetic variant is usually very small effect and hence the prior probability that a specific genetic variant interacts with a specific environmental factor is also very small. Therefore, the majority of published G × E studies have suffered from lack of replication, low-power, a publication bias towards positive results and major methodological concerns (for a critical review see Duncan & Keller, 2011). Interpretation of candidate gene G × E research is further complicated by the wide diversity of methods and reporting standards used, precluding meta-analytical evaluation of G × E evidence (Modinos *et al.* 2013). However, lack of power and replication was not limited to G × E studies but was also inherent in the decade of candidate gene association studies which were not powered to detect the small effect sizes that we now know operate in human diseases. Significant findings were almost never replicated and candidate genes that were selected based on their potential involvement in candidate biological pathways (e.g., neurotransmitter systems) have generally not shown robust association with psychiatric disease suggesting that current understanding of the biological basis of psychiatric disease is lacking.

The era of genome-wide association studies (GWAS) promised new hope but early studies were also underpowered (Manolio *et al.* 2009). More recent statistical analyses of aggregate single nucleotide polymorphism (SNP) effects, however, have shown that genetic variation in complex psychiatric disorders is polygenic in nature. Odds ratio of the individual variants generally range from 1.1 to 1.4; consequently, very large sample sizes are required to estimate those SNP effects with high precision. Recent efforts to increase sample sizes are now starting to pay off; the latest mega-analyses of GWAS data for schizophrenia has identified >100 genome-wide significant associations (2014), together explaining 7% of the variation in out of sample prediction. Other psychiatric diseases are expected to follow if GWAS sample sizes continue to increase.

Comparison of effect sizes of individual genetic risk variants with environmental risk factors shows that much larger risk can be attributed to individual environmental factors. For example, recent meta-analyses of the association between schizophrenia and urbanicity and migrant status revealed a pooled odds ratio of 2.39 for urbanicity (Vassos *et al.* 2012) and odds ratios of 2.7 and 4.5, for first and second generation migrants to European countries, respectively (Dealberto, 2010).

Taking these lessons forward to novel G × E research where we hypothesise that exposure to environmental risk factor increases the risk of disease only in those that are *genetically susceptible*, we will have to redefine being *genetically susceptible* in our study design. A single mutation in a candidate gene is unlikely to have a big impact in the population, by taking the aggregate effect of all mutations; however, we can differentiate people being at high genetic risk for disease. A polygenic architecture for psychiatric disorders of many weakly contributing variants means that genetic effects interact on the scale of disease (i.e., affected *v.* not affected) but act more additively on a susceptibility to disease scale (i.e., liability scale) (Zammit *et al.* 2010). On the disease scale, environmental variables are expected to combine interactively with genetic variants (combined), but more unknown is whether the genetic variants and environmental factors interact on the underlying scale where genetic effects combine more additively.

**Fig. 1.**
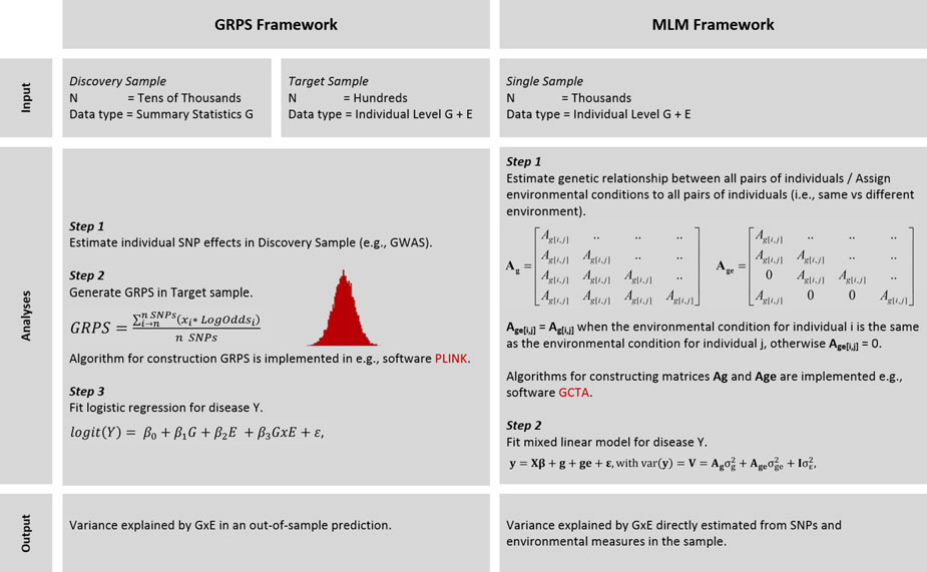
Summary of genetic risk profiling framework and mixed linear model framework for detecting and estimating G × E. GRPS, genetic risk profile score; MLM, mixed linear model; G, genetic condition; E, environmental condition; G × E, gene–environment interaction; **A**_**g**_, genetic relationship matrix; **A**_**ge**_, gene–environment relationship matrix; MLM, framework can also be applied in a bivariate setting in which the two traits represent the two environments; environmental conditions can be binary, ordinary and continues.

Here we describe two methods (see Fig. 1 for a schematic of the methods) that utilise the aggregate effect of genetic mutations to study potential G × E in a more powerful and reliable way compared with the candidate-gene approach.

## G × E in a risk-profiling framework

Genomic risk profile scores (GRPS) can be used as proxy estimate of genetic effects in a G × E study in which individuals have both genome-wide genotypes and measures for environmental risk factors. GRPS are quantitative scores calculated for each individual and are an estimate of an individuals' genetic risk for disease. GRPS were first applied to schizophrenia GWAS data where they provided evidence for a substantial polygenic component to the risk of schizophrenia involving many loci of very small individual effect (Purcell *et al.* 2009).

GRPS are calculated using estimates of genetic effect sizes derived from an independent GWAS ‘discovery sample’. It is important that no individuals (or their close relatives) from the GRPS sample (or ‘target sample’) are included in the discovery sample. GRPS are constructed in two steps: firstly, individual effect sizes of risk alleles (e.g., beta from linear regression, odds ratio from logistic regression or best linear unbiased prediction (BLUP) from linear-mixed models (Yang *et al.* 2014)) are estimated in the discovery sample. Secondly, for each individual within the target sample a GRPS is computed by taking the number of risk alleles an individual possesses weighted by the effect size of that allele from the discovery sample, averaged over the number of loci included in the GRPS.

The GRPS and the environmental variable of interest can now be included as individual terms as well as a product term in a risk-profiling framework. Fitting nested (increasingly more restricted) models allows testing for significance of the GRPS, the environmental factor of interest, and their interaction effect. For a disease trait (binary *Y*), the full logistic regression equation will be:

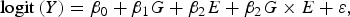

where *G* is the multi-locus GRPS, *E* is the environmental moderator variable and *G* × *E* is the interaction term between *G* and *E*. Under this model significant genetic and environmental effects already imply G × E on the disease (*Y*) scale, but a significant G × E term implies G × E on the underlying liability to disease. In this framework, multiple GRPS and multiple environmental factors can be included in the analyses to allow for hypothesis-driven identification of G × E effects. For example, GRPS can be based on SNPs that are selected based on functional annotation or physical position in the genome.

In the context of schizophrenia, the latest mega analysis published by the Psychiatric Genomics Consortium (PGC) (2014) (their Figure 3) shows that if individuals in independent target samples are ranked on GRPS then the odds of disease in the 10th decile is 7–20-fold (varying between samples) greater than the odds of disease in the first decile.

A G × E application may consider, for example, neonatal vitamin D level (McGrath *et al.* 2010*b*) as an environmental risk factor for schizophrenia that is hypothesised to interact with the GRPS. In this application, the G × E analysis could be extended by considering multiple GRPS and hence multiple genetic and G × E terms based on biological function, e.g., one based on SNPs in calcium ion channel genes (e.g., Purcell *et al.* 2014) and one based on the remaining SNPs.

Algorithms for the construction of GRPSs have been implemented in the software PLINK (http://pngu.mgh.harvard.edu/~purcell/plink/; option – score) (Purcell *et al.* 2007). The prediction accuracy of the constructed GRPS and the G × E effects can be analysed in a statistical software package of choice.

## G × E in a mixed linear model framework

The estimation of variance explained by aggregate SNP effects, sometimes called SNP heritability (see the Introduction section) is based on a mixed linear model framework. In this framework, genetic variance is estimated from genetic similarity among pairs of individuals who are not related in the classical sense. The basic idea behind this method is that for a polygenic trait, pairs of individuals who show higher genetic similarity also show higher resemblance at the trait level. Genetic similarity for each pair of individuals (defined in the model as sharing 0, 1 or 2 alleles at each locus) is measured from all the SNPs and the aggregated SNP effects are treated as random variables in a mixed linear model (Yang *et al.* 2010, 2011). The model can be augmented with G × E effects. Similarly to the SNP effects, the G × E effects are included as random effects in the model. In matrix notation, the linear mixed model including a G × E term can be written as:

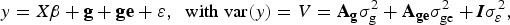

where *y* is an *n* × 1 vector containing the phenotypes (e.g., 1, affected *v.* 0, unaffected), **g**, **ge** and ***ε*** are vectors of length *n* of the aggregate effects of all the SNPs from all of the individuals, the genotype–environment interaction effects from all of the individuals, and the residual effects, respectively. The variance of *y* is the sum of the genetic variance, the interaction variance and the error variance. For example, when the environmental condition is binary, **A**_**g**_ is the genetic relationship matrix (GRM) estimated from all SNPs and elements in **A**_**ge**_ = **A**_**g**_ for all pairs of individuals sharing the same environment and elements in **A**_**ge**_ = 0 for the pairs of individuals in different environments.

The estimate of the aggregate SNP effects (**g**) reflects the genetic variation that is captured by common SNPs and the estimate of the G × E effects (**ge**) reflects the proportion of the variance attributable to G × E. Through application of restricted maximum likelihood estimation (REML), the proportion of genetic and/or G × E variance to the total variance can be estimated. Significance of the parameters can be tested by likelihood ratio tests comparing the likelihood under the full and reduced models.

When the *E* variable is binary, G × E can also be investigated using a bivariate mixed model with the two traits representing the two environments (Falconer & Latyszewski, 1952). Under this more general framework the SNP-heritabilities are not forced to be the same under the two conditions, an additional degree of freedom is however included in the model. A genetic correlation across the environments that is significantly less than one implies existence of G × E. However, when the SNP-heritabilities of the trait in the two environments differ a genetic correlation across environments that is equal to one does not necessary imply absence of G × E; in this scenario it is however most likely that the G × E term reflects a scale effect (Lynch & Walsh, 1998). To detect possible scale effects, data could be transformed prior to analysis; any variance that can be removed through transformation of the data can be labelled as a scale effect. Interactions reflecting scale effects can however not always be removed or even reduced by a transformation of scale (Falconer & Mackay, 1996).

For disease traits, interpretation of the estimates of the SNP-heritability of the two traits (i.e., heritability of the disease in the two environments) is problematic, exacerbated by potentially different lifetime disease prevalences under the two environmental conditions, which may be unknown or difficult to estimate. We advise to use the bivariate approach to test for significance of the G × E term, rather than interpreting the estimates of SNP-heritability in the two environments, since the correlation is not affected by the ascertainment imposed on the disease in the two environments. A magnitude of the interaction variance can be estimated in the univariate mixed linear model. Analysis under both the univariate and bivariate G × E frameworks is recommended to gain further insight into the estimated interaction.

Application of the mixed model method requires data sets in which all individuals are measured for genome-wide genotypes and the environmental risk factor. In large data sets the genetic and G × E terms can be partitioned by fitting multiple GRMs based on specific notations such as functional pathways, similar to fitting multiple GRPS × E interaction terms in the risk profiling framework.

Estimation of genetic variance and G × E variance in a linear mixed model has been implemented in the software GCTA (http://www.complextraitgenomics.com/software/gcta/) (Yang *et al.* 2011).

## Comparison of the two methods

The method of choice, risk-profiling or mixed linear model, primarily depends on the data available to researchers. The mixed linear model method requires large samples measured for both individual level genetic and environmental measures (based on power considerations (e.g., Dudbridge, 2013; Visscher *et al.* 2014), we estimate a sample size of at least 5000 individuals). Because individual studies are generally too small, researchers are likely to combine data from several studies into one study. The gain in sample size, however, often comes with a loss in coherence of both genetic and environmental measures. Recently developed methods in statistical genetics allow harmonisation of the genetic data, e.g., through imputation of SNP data to a common reference panel. Harmonisation of environmental measures across studies, however, needs more thoughtful discussion in the field, and new data collection efforts should aim for harmonisation with other studies (e.g., PhenX Toolkit; Hamilton & Tabitha, 2014).

In contrast, the risk-profiling method requires individual level genetic and environmental measures in only the target sample with the GRPS constructed based on GWAS summary statistics from a larger independent discovery sample that is (most likely) ignorant of the environmental conditions. Since the dearth of data sets informative for both genetic and environmental measures has been a limiting factor in G × E research, the risk-profiling framework is likely to be more widely applied and allows statistical power to be leveraged from larger samples not measured for the environment. In the risk-profiling framework, identification of true interaction effects largely depends on the prediction accuracy of the genetic and environmental factors. Prediction accuracy of GRPS is driven by precision with which the individual SNP effects are estimated in the ‘discovery sample’ in which large sample size generates higher precision. Combined efforts in the psychiatric genetics community (PGC) have achieved 34 241 schizophrenia cases, and 45 604 controls in 2014 (2014) resulting in high precision of the estimation of individual SNP effects and consequently large prediction accuracy of the GRPS. Precision of the environmental measures largely depends on the measure of interest. By nature, some environmental variables are measured with greater precision (e.g., migrant status) than others (e.g., age of first cannabis use). Large discovery sample sizes and well-defined environmental variables in the target samples will increase precision and prediction accuracy in the risk-profiling framework. Interpretation of results must consider the likely representation of unmeasured environmental risk factors in the discovery sample.

In both frameworks, the genetic architecture underlying the disease is a determinant in the statistical power of a study, this factor is however beyond our control. For example, larger sample sizes are required when common SNPs explain less variance (i.e., SNP heritability is low), which can be due to, for example, common SNPs being not in sufficient linkage disequilibrium (LD) with the causal variants or total heritability being low. In the risk-profiling framework, prediction efficacy (e.g., the amount of variance that can be explained by the GRPS) depends on the sample size of the discovery sample whereas the ability to detect variance explained that is significantly larger than zero depends on the size of the target sample. The same applies to variance explained by G × E. When sufficient samples are available and the researcher can apply both methods, the mixed linear model framework is to be preferred. It estimates the variance explained directly from individual-level genotype data, accounting for the correlation structure between the SNPs.

## Multi-locus G × E success

G × E studies that estimate genetic risk from genome-wide genotypes are in their early days since there are few data sets of sufficient size informative for both genetic and environmental factors. Much larger sample sizes, however, are expected to become available in the coming years allowing application of both frameworks to a wide variety of psychiatric diseases, either with direct measures of genetic risk and environment or through proxy-measures of both entities.

Using genetic summary statistics on alcohol problems in young adults from the Avon Longitudinal Study of Parents and Children (ALSPAC, *n* = 4304 individuals), Salvatore *et al.* (2014) show an association between the derived GRPS and alcohol problems in adolescents in an independent population based Finnish sample (FinnTwinn12, *n* = 1162). They also demonstrated interaction between the GRPS and two environmental factors: *parental knowledge* and *peer deviance*. Genetic factors related to alcohol problems were more pronounced under conditions of low parental knowledge and high peer deviance.

When environmental measures are not available in a case-control sample, association between genetic factors underlying the disease and the potential environmental moderator can be studied in samples with healthy individuals. Power *et al.* (2014) used GRPS for schizophrenia risk (Ripke *et al.* 2013), to explore the genetic relationship between schizophrenia and cannabis use in a population sample in which <1% would be expected to have lifetime schizophrenia. Cannabis use is well established to be much higher among schizophrenic patients compared with the general population, causality and its direction, however, is still under debate (e.g., Ferdinand *et al.* 2005; Green *et al.* 2005; McGrath *et al.* 2010*a*; Kuepper *et al.* 2011). GRPS for schizophrenia were associated with cannabis use (*ever v. never* as well as *quantity of use*) in a sample of 2082 healthy individuals. This result does not exclude the possibility of a causal relationship but shows that at least part of the association between schizophrenia and cannabis use may be due to a shared genetic aetiology.

An approach to study G × E when measures on environmental risk factors are not directly available is to use epigenetic markers as proxies for the environment. Epigenetic markers associated with for example smoking behaviour (Shenker *et al.* 2013; Zeilinger *et al.* 2013) can be included as proxy-environmental moderators in the model both as a main effect and in an interaction term with genetic risk.

An example of a G × E study in a linear mixed model framework is a bivariate analysis of schizophrenia in which the two traits represent two different populations: European and African descent (de Candia *et al.* 2013). The genetic correlation derived from SNP similarity within and between populations was estimated at 0.66 (s.e. = 0.23) and was significantly different from zero but not from one. The results were not suggestive of G × E interaction and suggested that many schizophrenia risk alleles are shared across ethnic groups.

## Conclusion

The goal of G × E research now and in the near future is the identification of novel genetic pathways that do not have marginal effects and the discovery of environmental risk factors that affect only a subpopulation of genetically susceptible individuals. Increasing sample sizes in psychiatric genetics research are starting to show that genetic risk predictors could have utility for stratification of individuals into high- and low-risk groups for developing disease (PGC–SCZ, 2014). Augmenting these genetic risk predictors with environmental moderators should increase prediction accuracy. Diagnostic use of multi-locus genetic risk predictors is a long-term goal that might come closer once informed by environmental predictors. Real progress in G × E research requires concerted effort of collection of informative data sets.
